# Familial context influences media usage in 0- to 4-year old children

**DOI:** 10.3389/fpubh.2023.1256287

**Published:** 2024-01-11

**Authors:** Frank W. Paulus, Jens Joas, Anna Friedmann, Tamara Fuschlberger, Eva Möhler, Volker Mall

**Affiliations:** ^1^Child and Adolescent Psychiatry, Saarland University Hospital and Saarland University Faculty of Medicine, Homburg, Germany; ^2^Technical University of Munich, TUM School of Medicine and Health, Chair of Social Pediatrics, München, Germany

**Keywords:** parental media usage, children’s media usage, family factors, Problematic Internet Use, familial context, young children

## Abstract

**Background:**

The use of digital media (e.g., smartphones, tablets, etc.) and the Internet have become omnipresent for every age group and are part of children’s and parents’ everyday life. Focusing on young children, the availability of media devices, their use as well as associated problems (e.g., in social, emotional and motor development) have increased in recent years. Of particular interest for prevention of these problems in early childhood is the relationship between the familial context (parental digital media use, Problematic Internet Use, school graduation, presence of siblings) and the digital media use of infants and toddlers. The present study’s goal was to describe media usage in 0–4-year-old children and to identify the potential relationship between familial context factors and child media usage.

**Methods:**

The sample included *N* = 3,035 children aged 0 to 3;11 years (*M =* 17.37 months, *SD* = 13.68; 49.13% female). Recruitment took place within the framework of a restandardization study for a German developmental test. The parents of the participants answered a questionnaire on socio-demographics, on child media use, and on parental media use. Questions on parental media use included the full version of the Short Compulsive Internet Use Scale (S-CIUS).

**Results:**

Significant increases in media usage times with child age were identified, but no significant gender differences. A multiple regression analysis revealed that increasing maternal total media usage time, a higher parental S-CIUS score, lower school leaving certificate of both mother and father, and increasing child’s age led to higher child media usage time. Having siblings diminished young children’s media usage in this study. Having more than one child and having children aged over a year was associated with a higher parental S-CIUS score.

**Conclusion:**

Family factors such as maternal media use time, Problematic Internet Use and lower school graduation are significantly associated with young children’s digital media use. Parents should be aware of their personal influence on their children’s media use which might be due their role in terms of model learning.

## 1 Introduction

These days, children and adolescents grow up in homes with media like television, smartphones, computers, tablets, smart watches and gaming consoles being highly present and used, further reinforced by the COVID-19 pandemic (1–9). Due to the progressing digitization, children and adolescents as well as their parents, caregivers, teachers, therapists and doctors are being confronted with new issues and disorders arising from this development like Gaming Disorder (10–13), Internet Addiction or Problematic Internet Use (14–16). As nosology currently cannot keep up with the rapid technological development of digital hardware and applications over the last two decades, literature uses various terms for describing this clinical entity. This results in a multitude of different and partly conflicting conceptualizations of digitization-related disorders with different diagnostic criteria and test procedures.

Excessive media usage can influence a child’s or adolescent’s development in a way that prevents usual developmental tasks or milestones from being reached. The foundations for functional or dysfunctional and impairing media consumption are not laid in adolescence or childhood, but in preschool, toddlerhood and infancy. Especially social, emotional, cognitive, verbal and motor skill development as well as nutrition and sleep are negatively affected by early digital media usage (17–23). Time spent with using digital media devices can displace the time usually spent with parents or other family members (24) and result in multiple negative consequences (e.g., impaired language and executive functions, impaired caregiver-child relationship, anxiety, behavioral difficulties, cardiovascular risk) especially for infants and preschool children (25–29). Additionally, an increasing amount of parents are using mobile devices as distractions while with their children, resulting in a lack of parenting responsiveness and quality (30, 31). This leads to the assumption that „digital native“ parents are engaging in media use behaviors that affect their children’s development, as well as their own sensitivity (32, 33) toward their child, especially in the first year of life. Additionally, parental media use during parent–child-interactions (technoference) may influence the child’s externalizing and withdrawal behavior (34) and may lead to “maladaptive technological behaviors” (35).

Currently, the age at which children start using media is shifted to preschool age and infancy (24), partly because of the new interactive media devices (36) accompanied by touch screens’ simplified handling and voice control (37). As a result of market development and technical innovations, usage times have skyrocketed, with young children being specifically and more intensely targeted as consumers.

In order to be able to possibly prevent or reduce young children’s media usage, it is essential to understand which contextual conditions contribute to this problem. Models that aim to explain the development of Gaming Disorder, Problematic Internet Use or other disorders that are related to digitization are multicausal and include internal factors like structural and functional neurobiological abnormalities, executive disorders and comorbid psychological disorders as well as external (parental modeling of how to interact with media) and social factors (family’s socioeconomic situation) (12, 38–40).

Since young children are reliant on their parents for a plethora of things it makes sense to investigate the familial context when addressing influences on children’s usage of media and screen time.

Generally, children’s media usage patterns have been reported to be similar to their parents’ (41): parents who consume a large amount of media themselves are more likely to raise children who are exposed to and use media early on than parents with a more reserved approach to media usage.

Parents’ socioeconomic status has been linked to young children’s media usage: Children in lower educated, lower income families are reported to have more devices in their bedroom and spend more time using media than children whose parents have a higher socioeconomic status (41–44).

Looking at parents separately as individuals, several studies take the mother’s education into account [e.g., (45, 46)]. Rideout and Hamel (43) report that young children with mothers who have not finished a high school education spend more time in front of a screen on a daily basis than children whose mothers have obtained a higher level of education. In line with these findings, Anand and Krosnick (47) found that mothers’ lower education resulted in more TV watching in children between 6 months and 6 years, with the same result found for fathers. Hoyos and Jago (48) report that both parents’ common education level is moderately negatively associated with screen-time while fathers’ education level shows a strong negative correlation with children’s screen-time.

While some research suggests that young children who have siblings are more likely to engage in daily media use than only children are (49), other research has not been able to replicate these effects (41). Children with siblings as well as their families might engage in more activities that are alternatives to media and screen time than families with only children do. This could imply that children who do not have siblings might spend comparatively more time using media and more time in front of screens than young children who have siblings. The effect that having siblings may or may not have on young children’s media usage is one that has been yielding inconclusive results. De Decker et al. (50) conducted a qualitative interview-study in 6 European countries and found, that parents in Bulgaria, Germany and Spain believe that siblings or friends have a major influence on children’s screen time whereas the attitudes of parents from Greece, Poland and Belgium were inconclusive. The conflicting findings found in the literature may be due to the influence of the age of siblings, as older siblings might be seen as role models and might have a stronger influence on the media usage behavior than siblings of the same age. Moreover, gender differences could also influence the relationship between siblings and digital media use, as mentioned by Bagley et al. (51).

In line with developmental progress, age overall is strongly positively associated with screen time in young children (48). Older children are reported to have a higher media consumption than younger children and it can be considered confirmed that a child’s age generally is a significant predictor of their usage of media (47).

With regard to gender differences, studies note that there is a preference for gaming among boys and a preference for social media use among girls (52, 53). Regarding younger samples, Green et al. (54) also found gendered differences in the time spend on video game usage. In a longitudinal study spanning 3 years with children of the ages 2 and 4 at the start, they found that boys spend more time playing video games than girls and that these differences increase with age. In line with this finding, a nationwide survey conducted by Ofcom in the United Kingdom in 2014 (55) showed that 30% of boys aged 3 to 4 use a games console, but only 21% of girls aged 3 to 4. The miniKIM-Studie (6) however does not find any significant gender differences in two- to five-year-old children. It is to be explored whether gender differences may not yet be so pronounced at this early age. Findings on gender differences in younger children and infants are lacking, as is research on gender differences in general digital media use time in this age group.

All these afore mentioned factors have been shown to have some effect on children’s and adolescent’s media behavior. However, it is still unclear in many ways to what extent this applies for young children as well. In addition, reciprocal relationships between children’s and parents’ media use could also be possible, in the sense that even young children could have an influence on parental media use. Obtaining more data seemed necessary to identify patterns that might result in or from young children’s media usage.

Therefore, in this study, we hypothesize that media usage in children aged 0 to 4 is predicted by familial context. More specifically, our first hypothesis is that parents’ increased media usage time and parents’ Problematic Internet Use are positively correlated with their young children’s time spent using media. An associated research question to be answered by this study is whether there is a reciprocal relationship between child characteristics and parental media use in the sense that child variables could influence parental media use, too. Hypothesis 2 states that parents level of education is a predictor for the amount of time children use media: higher level of education is associated with less time using digital media. The third hypothesis postulates that a child’s age positively predicts their media usage time: The older the child, the more it uses digital media.

Concerning the mixed results regarding siblings and their influence in research so far, a research question we aim to answer in this study is how the presence of siblings affects young children’s media usage.

## 2 Materials and methods

### 2.1 Study design

The sample was recruited within the framework of a restandardization study for the Münchner Funktionelle Entwicklungsdiagnostik (MFED), consisting of a prospective cross-sectional study. The preparation for the restandardization study project (MFED) started in 2015. The associated media study reported in this paper was prepared from January 2019, and data collection took place from May 2019 to March 2022. This study is monocentric, being conducted by the Chair of Social Pediatrics at the Technical University of Munich, and the kbo Kinderzentrum München.

The aim of the study was to carry out the investigations throughout Germany. The distribution is as follows: 58.6% Bavaria, 21.0% Berlin, 5.6% North Rhine-Westphalia, 4.9% Baden-Württemberg, 2.5% Thuringia, 2.4% Saarland, 2.0% Saxony-Anhalt, 1.3% Lower Saxony, 0.8% Bremen and 0.3% Saxony. 0.8% of the children were examined in Innsbruck (Austria).

Participating families were recruited in pediatrician’s practices, hospitals, daycare centers/preschools, and through the distribution of flyers in playgrounds, counseling centers, etc. The children, accompanied by their parents, were invited to participate in the study by the examiners.

The questionnaire was completed by the parents at home or during the child’s developmental examination. All participants’ parents were informed and asked for written consent for participation in the study.

### 2.2 Participants

The sample used for our study included children aged from a few days postnatal age to 3 years and 11 months whose development had been normal up to that point.

Exclusion criteria were as follows: prematurity (birth weight under 1,500 g), a mother tongue different from German, medication impacting children’s cognitive or verbal performance as well as sensory or motor disabilities. Illnesses with a heightened risk of developmental disorders or genetic disorders were also excluded. No people who in any way were dependent on the director of studies or doctor/scientist responsible for this study were included.

**Figure 1:**
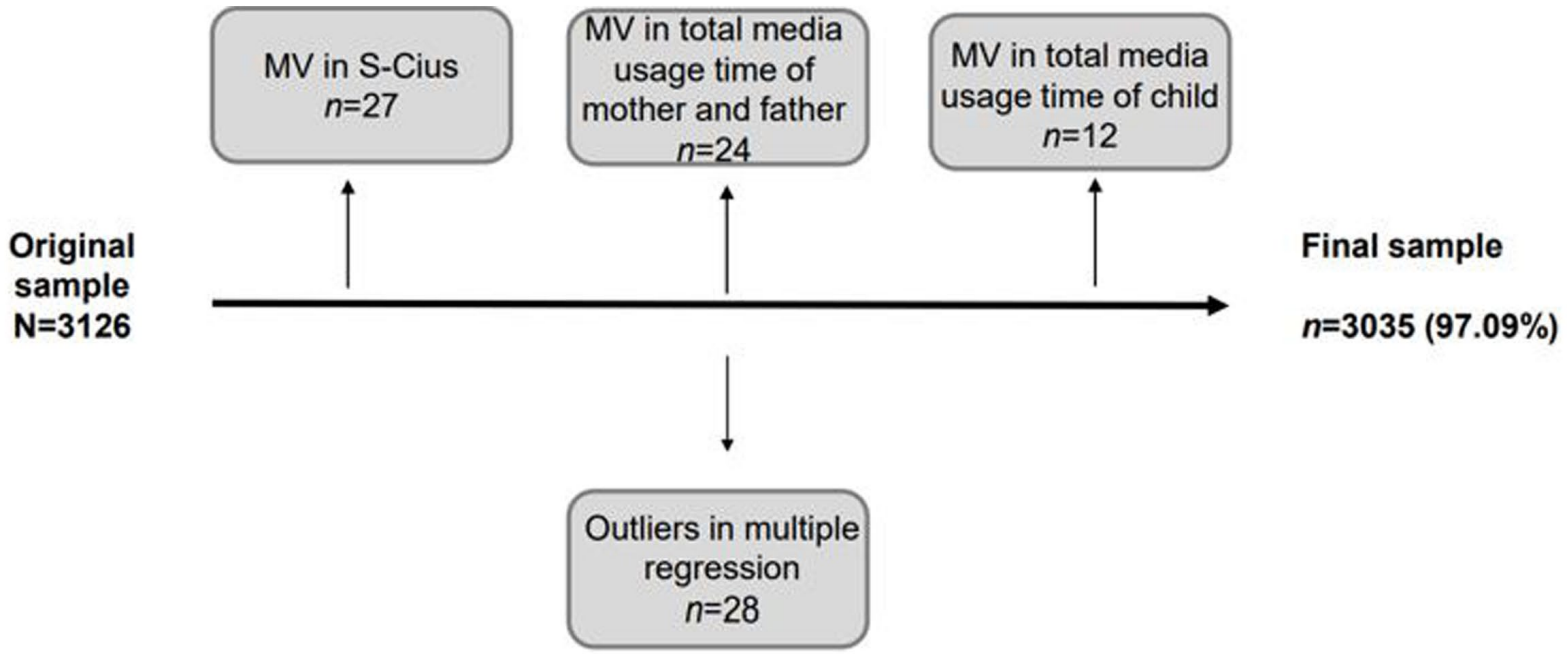
Flow of participants. MV, missing value. Participants were excluded as outliers in the multiple regression analysis (3 *SD* or more, based on standardized residuals).

The original *ad-hoc* sample consisted of 3,126 children. 12 (0.38%) had to be excluded because of missing values in total media usage time, 27 (0.86%) had missing values in S-CIUS and therefore became ineligible for further analysis and 24 (0.77%) children had to be excluded because of missing values in the total media usage time of their mother and father. Lastly, 28 (0.90%) data sets were excluded as outliers (participants were excluded as outliers in the multiple regression analysis (3 *SD* or more, based on standardized residuals)), so the final sample consists of 3,035 participants, 97.09% of the original sample. The participants flow can also be found in Figure 1.

### 2.3 Measures

The questionnaire on socio-demographics and on media usage, times of use and contexts of use of children aged 0–4 years and their parents is a questionnaire developed by the Child and Adolescent Psychiatry, Saarland University Hospital in cooperation with the Technical University of Munich in 2019. The questionnaire contains 57 items and was designed to assess general information, such as demographic information, school leaving graduation of mother and father as well as leisure activities and contexts of use and times of use of electronic media (e.g., television, computer/laptop, smartphone, smartwatch, tablet, game console) in children and parents.

Information about the child (10 items; e.g., gender, number of siblings, position of the child in the family, illnesses) and the child’s living circumstances (1 item, single-choice; e.g., living with both biological parents; see Table 1) are asked. Furthermore, the questionnaire asks whether the child attends a nursery/kindergarten and whether digital media are used there (dichotomous response format; yes/no), whether the child is in a club, and which activities the child likes (open response format). The questionnaire asks which media devices are available in the household (e.g., Smarttoy; see Table 2), which devices the child uses on a daily basis (open response format) and looks at how much time is spent with them (on average per day; indicated in minutes; see Table 3) as well as in which contexts (e.g., for the child’s occupation, at mealtimes, during waiting times, etc.; see Table 4). The questionnaire also asks whether the child can freely dispose of his or her media time, whether he or she has free access to the internet and whether safety locks have been installed (dichotomous response format; yes/no).

**Table 1:** Sample characteristics.

Characteristic	*M*	*SD*
**Siblings**	***n***	**%**
Single child	1,345	44.32
Has siblings	1,690	55.68
1	1,198	39.47
2	374	12.32
3	76	2.50
4	30	0.99
≥ 5	12	0.40
Multiple birth	137	4.51
**Attends nursery/Kindergarten**	1,055	34.76
**School graduation mother**	***n***	**%**
Without a school-leaving certificate	23	0.76
Special school certificate	5	0.16
Secondary school	217	7.15
Intermediate Secondary School Certificate (MSA)	750	24.1
High school	531	17.50
University	1,507	49.65
MV	2	0.07
**School graduation father**	***n***	**%**
Without a school-leaving certificate	30	0.99
Special school certificate	8	0.26
Secondary school	390	12.85
Intermediate Secondary School Certificate (MSA)	671	22.11
High school	390	12.85
University	1,500	49.42
MV	46	1.52
**Place of residence of child**	***n***	**%**
Lives with mother and father	2,843	93.67
Lives with mother	150	4.94
Lives with father	1	0.03
Lives with mother and her new partner	27	0.89
Lives with father and his new partner	2	0.07
Does not live with biological parents (e.g., foster family, adopting parents)	12	0.40
**Questionnaire answered by**	***n***	**%**
Mother alone or with another person (e.g., father, new partner)	2,835	93,40
Father	194	6.39
Other (e.g., grandparents)	6	0.20

MV, missing value.

**Table 2:** Media characteristics by household and child.

Characteristic	Child			
Media devices available in the household	*n*	%	MV	
			*n*	%
Smartphone	2,836	93.44	0	0
TV	2,657	87.55	0	0
Laptop	2,552	84.09	0	0
Tablet	1866	61.48	0	0
Console	748	24.65	0	0
Alexa	436	14.37	0	0
Smartwatch	398	13.11	0	0
Smarttoy	70	2.31	0	0
Other	182	6.00	0	0

Media user or no media user	*n*	%	MV	
			*n*	%
No media user	1,457	48.01	0	0
Media user	1,578	51.99	0	0

Internet access child	*n*	%	MV	
			*n*	%
Free access to the internet	4	0.13	15	0.49
Internet child safety lock installed	634	20.89	23	0.76

Media use in nursery/Kindergartens	*n*	%	MV	
			n	%
Total (*n* = 1,055)	64	6.07	7	0.66
Up to 1-year-olds (*n* = 12)	0	0	0	0
1-2-year-olds (*n* = 251)	8	3.19	0	0
2-3-year-olds (*n* = 361, MV = 2 = 0.55%)	18	4.96	2	0.55
3-4-year-olds (*n* = 424, MV = 5 = 1.17%)	38	8.86	5	0.66

MV, missing value; No media user means: parents indicated a daily use of 0 min for their child for all digital media devices indicated.

**Table 3:** Frequency and percentage of children’s media use and child average daily media usage time by age, gender, and siblings present or missing.

Child media use	Total	Up to 1 year old	1 to 2 years old	2 to 3 years old	3 to 4 years old	Male	Female	Single child	Has siblings
***N***	3,035	1,388	703	487	457	1,544	1,491	1,345	1,690
***n* %**	100	45.73	23.16	16.05	15.06	50.87	49.13	44.32	55.68
**No media user *n***	1,457	1,134	271	41	11	745	712	731	726
**No media user %**	48.01	81.70	38.55	8.42	2.41	48.25	47.75	54.35	42.96
**Media user *n***	1,578	254	432	446	446	799	779	614	964
**Media user %**	51.99	18.30	61.45	91.58	97.59	51.75	52.25	45.65	57.04
**from here on, all data refer to media users only:**									
**Digital gaming user *n***	47	0	4	15	28	27	20	19	28
**Digital gaming user %**	2.98	0	0.93	3.36	6.28	3.38	2.57	3.09	2.90
**No digital gaming user *n***	1,531	254	428	431	418	772	759	595	936
**No digital gaming user %**	97.02	100	99.07	96.64	93.72	96.62	97.43	96.91	97.10
Child (Video-) Calling/Skype time	1.79	2.25	2.62	1.43	1.10	1.69	1.90	2.42	1.39
Child internet time	0.09	0.00	0.01	0.20	0.10	0.10	0.08	0.07	0.10
Child movies/series time	16.88	4.39	9.89	21.23	26.41	16.56	17.20	12.61	19.59
Child digital games time	0.45	0.00	0.06	0.63	0.90	0.59	0.31	0.58	0.37
Child digital picture books time	0.33	0.00	0.37	0.16	0.67	0.23	0.44	0.24	0.40
Child other media time	1.11	0.55	1.43	1.25	0.97	0.95	1.26	1.45	88
**Child total screen time**	**20.65**	**7.20**	**14.38**	**24.90**	**30.14**	**20.13**	**21.18**	**17.38**	**22.73**
Child music/audiobook time	19.19	21.31	20.75	18.82	16.85	19.28	19.11	22.35	17.18
**Child total media usage time**	**39.84**	**28.51**	**35.13**	**43.71**	**46.99**	**39.40**	**40.29**	**39.73**	**39.91**

No media user means: parents indicated a daily use of 0 min for their child for all digital media devices indicated; Total screen time = screen time spend with (Video-) Calling/Skype + Internet + movies/series + digital games + apps + digital books and newspapers; Total media usage time = Total screen time + music/audiobook; All times are in minutes.

**Table 4:** Frequency and percentage of children’s use of electronic media in different contexts (overall and separated by the first 4 years of life) in parental judgment.

Contexts of use of electronic media in parental rating	Total (*n* = 1,578)		Up to 1 year old (*n* = 254)		1 to 2 years old (*n* = 432)		2 to 3 years old (*n* = 446)		3 to 4 years old (*n* = 446)	
	*n* = 1,072 (MV = 506)	67.93% (MV = 32.07%)	*n* = 111 (MV = 143)	43.70% (MV = 56.30%)	*n* = 278 (MV = 154)	64.35% (MV = 35.65%)	*n* = 327 (MV = 119)	73.32% (MV = 26.68%)	*n* = 356 (MV = 90)	79.82% (MV = 20.18%)
At mealtime	65	6.06	5	4.50	19	6.83	29	8.87	12	3.37
Before bedtime	304	28.36	30	27.03	65	23.38	96	29.36	113	31.74
To occupy/calm the child	403	37.59	58	52.25	119	42.81	112	34.25	114	32.02
During waiting times	205	19.12	11	9.91	48	17.27	71	21.71	75	21.07
When parents have no time (e.g., doing chores etc.)	480	44.78	18	16.22	100	35.97	153	46.79	209	58.71
With other children	160	14.93	17	15.32	65	23.38	45	13.76	33	9.27
**Total**	**1,617**	**150.84**	**139**	**125.23**	**416**	**149.64**	**506**	**154.74**	**556**	**156.18**

For media users only; Multiple answers are possible; MV, missing value.

Information about the highest school-leaving graduation (e.g., High School, University; see Table 1) of the biological parents are asked.

Additionally, the parents’ media consumption is recorded in detail, in particular how much time mother and father spend per day on average with different screen media (see Table 5). The Short Compulsive Internet Use Scale (S-CIUS) (56) is a short form of the Compulsive Internet Use Scale (CIUS) (57) and embedded in the above-mentioned questionnaire. It’s a screening tool to assess Problematic Internet Use (PIU). It consists of 5 of the original 14 items rated with a five-point Likert scale. The items are as follows: “How often do you find it difficult to stop using the internet when you are online?’’, “How often do other people (parents, friends) say you should use the internet less?’’, “How often do you sleep too little because of the internet?’’, “How often do you neglect your daily obligations because you prefer to go online?’’ and “How often do you go online when you are feeling down?’’. The response options for each are “0 = never, 1 = seldom, 2 = sometimes, 3 = frequent, 4 = very frequent’’. Its reliability of 0.77 (Cronbach’s Alpha) is adequate. At a cut-off of 7 which was shown to perform best in case detection, it yields a sensitivity of 0.95 and a specificity of 0.87 (58). In all these psychometric properties it is no worse than its full-length version.

**Table 5:** Frequency and percentage of media use by mother and father and average daily media usage times.

Characteristic	Mother			Father		
Media user or no media user	*N*	%	MV	*n*	%	MV
No media user	22	0.72	5 (0.16%)	118	3.89	114 (3.76%)
Media user	3,008	99.11		2,803	92.36	

From here on, all data refer to media users only:										
Media usage time	*M*	*SD*	Min.	Max.	MV	*M*	*SD*	Min.	Max.	MV
(Video-) Calling/Skype	14.09	26.95	0	480	0	22.21	52.44	0	510	0
Internet	47.51	45.65	0	480	0	57.29	59.14	0	720	0
Movies/series	53.20	48.62	0	480	0	56.83	48.42	0	360	0
Digital games	2.49	12.21	0	240	0	12.08	30.46	0	420	0
Apps	24.50	30.76	0	420	0	22.98	30.82	0	300	0
Digital books and newspaper	9.82	20.29	0	300	0	14.71	25.09	0	300	0
Other	7.96	49.68	0	510	2	54.36	138.25	0	720	0
**Total screen time**	**159.57**	**105.90**	**0**	**990**	**0**	**240.46**	**194.61**	**0**	**1,290**	**0**
Music/audiobooks	33.11	61.00	0	960	0	28.47	57.21	0	960	0
**Total media usage time**	**192.68**	**132.21**	**3**	**1,080**	**0**	**268.92**	**212.18**	**7**	**1,500**	**0**

No media user means: parents indicated a daily use of 0 min for all digital media devices indicated; Total screen time = screen time spend with (Video-) Calling/Skype + Internet + movies/series + digital games + apps + digital books and newspapers; Total media usage time = Total screen time + music/audiobook; All times are in minutes; MV, missing values.

### 2.4 Statistical analysis

The contents of the study were evaluated descriptively and via inferential statistics. A multiple regression analysis was conducted to predict the child’s total media usage time. To compare the effect of age group (children) on Problematic Internet Use (S-CIUS total score) (parents) a one-way ANOVA was performed. Because of the violation of the preconditions (such as homoscedasticity and normal distribution) and unequal group sizes, a Brown-Forsythe ANOVA was calculated. Additional t-tests were performed for group analysis of continuous variables. Since the requirements for a t-test for independent samples were not met, a Mann Whitney U Test was calculated.

Data were analyzed using the IBM SPSS Statistics version 26. A significance level of 0.05 was used for all statistical tests.

## 3 Results

### 3.1 Descriptive analysis

#### 3.1.1 Sample

The participants’ mean age was 17.37 months (*SD* = 13.68, Min. = 0, Max. = 47) with 1,544 (50.87%) of them being male and 1,491 (49.13%) being female. The average maternal age at birth was approximately 32 years (*SD* = 4.73, Min. = 14, Max. = 52). The average paternal age at birth 35.16 years (*SD* = 5.76, Min. = 15, Max. = 67). 93.40% of the questionnaires were filled out by the mother alone or by the mother with another person (e.g., father/new partner). At the time of data collection 2,843 (93.67%) children lived with both of their biological parents. In case of the biological parents being split up, most children lived with their mothers and not their fathers. Only 10 (0.33%) lived in foster families or with adoptive parents. 1,690 (55.68%) had at least one sibling while 1,345 (44.32%) were only children. 2038 of the mothers (57.15%) had graduated university or a finished high school education, 31.25% finished secondary or intermediate secondary school, and 0.92% had none or a special school certificate. Among fathers, 62.27% had graduated university or a finished high school education, 34.96% finished secondary or intermediate secondary school, and 1.25% had none or a special school certificate (see Table 1).

#### 3.1.2 Media characteristics by household and child

The *media devices available in the household* can be found in Table 2. The most owned items used for consuming electronic media among families were smartphones (93.44%), televisions (87.55%), laptops (84.09%) and tablets (61.48%). Consoles were present in 24.65% of households.

Tables 2, 3 show the children’s media behaviors, such as total *daily media usage time* in minutes as well as daily media usage time categorized by media type. Out of 3,035 children, about half of them were reported not to be media-users (= parents indicated a daily use of 0 min for their child for all digital media devices indicated) (48.01%). The other 51.99% of children use electronic media for an average of 39.84 min (*SD* = 34.30, Min. = 1, Max. = 300) per day, of which about half is spend on screens (20.65 min; total screen time is defined as the sum of screen time spend with (Video-)Calling/Skype, Internet, movies/series, digital games, apps and digital books and newspaper). The following usage times occur among the media users: Most popularly used by children are music and audiobooks (19.19 min/day), followed by movies and series (16.88 min/day). Least used were the Internet (0.09 min/day) and digital picture books (0.33 min/day). As can be seen in Table 3, a children’s average total media usage time per day increases with their age. There were no missing values for the media characteristics by child.

Only 0.13% of children have free access to the Internet and 20.89% of households have a child safety lock installed.

If we look at *media use in the nursery/kindergarten*, we see that on average 6.07% of those children who attend a nursery/kindergarten also consume media there. However, children under 1 year of age do not consume media in the nursery/kindergarten, the number of consumers then increases across the age groups and reaches 8.86% among the 3- to 4-year-olds.

##### 3.1.2.1 Age differences

Among children who use digital media, total digital media usage time averages 28.51 min per day in the first year of life, 35.13 min per day in the second year of life, 43.71 min per day for 2- to 3-year-olds and 46.99 min per day for 3- to 4-year-olds. Due to the presence of heteroskedasticity [Levene’s *F* (3, 3,031) = 184.83, *p* ≤ 0.001], lack of a normal distribution and unequal group sizes, a Brown-Forsythe ANOVA was performed. This showed that there was a statistically significant difference in children’s overall media use time between at least two age groups [Brown-Forsythe- *F* (3, 3,031) = 360.69, *p* ≤ 0.001, *n* = 3,035]. The estimated ω^2^ = 0.26 indicates a large effect. Games-Howell post-hoc procedure showed that the mean value of children’s media use differed significantly between all age groups.

The number of those who use *digital games* is increasing rapidly with age: while no digital games are used among the under-one-year-olds, the number of users doubles from the third to the fourth year of age (2- to 3-year-olds: 3.36%; 3- to 4-year-olds: 6.28%). While under-one-year-old media users watch *movies/series* an average of 4.39 min per day, the 1-2-year-olds increase it to 9.89 min, the 2-3-year-olds to 21.23 min and the 3-4-year-olds to 26.41 min.

A similar increase is seen in *total screen time* (up to 1 year old: 7.20 min; 1 to 2 years old: 14.38 min; 2 to 3 years old: 24.90 min; 3 to 4 years old: 30.14 min). Due to the presence of heteroskedasticity [Levene’s *F* (3, 3,031) = 338.50, *p* ≤ 0.001], lack of normal distribution and unequal group sizes, a Brown-Forsythe ANOVA was performed. This showed that there was a statistically significant difference in children’s screen time between at least two age groups [Brown-Forsythe- *F* (3, 3,031) = 438.02, *p* ≤ 0.001, *n* = 3,035]. The estimated ω^2^ = 0.30 suggests a large effect. Games-Howell post-hoc procedure showed that the mean value of children’s screentime differed significantly between all age groups.

Time spent with music and audio books decreases with age (up to 1 year old: 21.31 min; 3 to 4 years old: 16.85 min). The time spent with ((Video-)Calling/Skype), internet and digital games hardly changes over the age range considered here. Children under the age of one do not use the internet. If we look at single children versus siblings, children with siblings are more often electronic media users (57.05%) than single children (45.65%). A chi-squared test confirmed that the percentage of electronic media users did differ by existence of siblings χ^2^(1, 3,035) = 38.93, *p* ≤ 0.001. We see in particular that siblings spend more time watching films and series (19.59 min versus single children, 12.61 min).

##### 3.1.2.2 Gender differences

In our sample, there are no *gender differences* between users of electronic media: 51.75% of male and 52.25% of female children are users. Looking at gender differences (media users only), boys (*M* = 0.59 min, *SD* = 4.23) played on average longer *games* than girls (*M* = 0.31 min, *SD* = 2.33). According to Mann Whitney U test, however, this was not a significant difference (*U*(*n* boys =799, *n* girls = 779) = 308644.00, *z* = −0.96, *p* = 0.34).

Girls (*M* = 0.44, *SD* = 3.21) read more digital picture *books* than boys (*M* = 0.23, *SD* = 2.90). However, according to Mann Whitney U test, this was not a significant difference (*U*(*n* boys = 799, *n* girls = 779) = 309810.00, *z* = −0.62, *p* = 0.53).

Girls (*M* = 21.18, *SD* = 23.49) had a higher *screen time* than boys (*M* = 20.13, *SD* = 23.06). Again, according to Mann Whitney U test, this was not significant *U*(*n* boys = 799, *n* girls = 779) = 299664.00, *z* = −1.29, *p* = 0.20. Girls (*M* = 40.29, *SD* = 34.43) also had higher average daily *media usage times* than boys (*M* = 39.40, *SD* = 34.19) but also, the difference was not significant according to Mann Whitney U test (*U*(*n* boys = 799, *n* girls = 779) = 310476.00, *z* = −0.81, *p* = 0.94).

#### 3.1.3 Media characteristics by parents

The vast majority of parents use digital media (mothers: 99.11%; to a slightly lesser extent fathers: 92.36%; see Table 5). The following usage times occur among the media users. Mothers spend on average 192.68 min a day using various media. Looking more closely at the mothers’ usage time, on an average day 159.57 min are spent on screen media, 53.20 min on watching films and series and 47.51 min on the internet. Fathers spend a daily average of 268.92 min using media, 240.46 of those on screens. Leading among fathers were movies and series as well as the Internet, each with about 57 min per day.

Looking at the S-CIUS scores (see Table 6), in total, 356 parents (11.73%) had a result above the cut-off 7, which implies Problematic Internet Use. Overall, the average total value was 3.03 (see Table 6).

**Table 6:** Descriptive statistics of S-CIUS values (parents) total and by child age, child gender, and siblings present or missing.

Items S-CIUS (parents)	Total	Up to 1 year old	1 to 2 years old	2 to 3 years old	3 to 4 years old	Male	Female	Single child	Has siblings
*M*	3.03	2.65	3.25	3.50	3.38	3.01	3.06	2.89	3.15
*SD*	2.78	2.59	2.90	2.89	2.91	2.77	2.81	2.76	2.80
Minimum	0	0	0	0	0	0	0	0	0
Maximum	16	14	16	13	13	16	14	16	15
Cut-off S-CIUS	≥ 7								
*n* ≥ cut-off	356	124	94	71	67	179	177	148	208
% ≥ cut-off	11.73	8.93	13.37	14.58	14.66	11.59	11.87	11.00	12.31

#### 3.1.4 Reciprocal relationship between familial factors and media usage

*Parents of multiple children* (*M* = 3.15, *SD* = 2.80) scored higher in S-CIUS than parents of only children (*M* = 2.89, *SD* = 2.76). A Mann Whitney U Test indicated that this difference was statistically significant (*U*(*n* multiple children = 1,690, *n* single child = 1,345) = 1069973.50, *z* = −2.80, *p* ≤ 0.01). The effect size according to Cohen (59) is Pearson *r* = 0.05 and is below a small effect (*r* = 0.10).

The S-CIUS score of parental media use also differed depending on the *age of the child*. In the first year of life, the parents’ S-CIUS total score was 2.65 (8.93% above the Problematic Internet Use PIU cut-off), in the second year of life 3.25 (13.37% PIU), in the third year of life 3.05 (14.58% PIU) and in the fourth year of life 3.38 (14.66% PIU). An ANOVA with the 4-fold stepped factor age was calculated on the S-CIUS total values. Due to heteroskedasticity [Levene’s *F* (3, 3,031) = 8.45 *p* ≤ 0.001], lack of normal distribution and unequal group sizes a Brown-Forsythe-ANOVA was performed. This revealed that there was a statistically significant difference in terms of Problematic Internet Use (S-CIUS total score) between at least two groups [Brown-Forsythe-*F*(3, 3,031) = 17.53, *p* ≤ 0.001, *n* = 3,035]. The estimated ω^2^ = 0.02 suggests a small effect. Games-Howell post-hoc procedure revealed that the mean S-CIUS total score differed significantly between parents of infants up to 1 year old and parents of all other age groups [compared to 1–2 year old infants *p* ≤ 0.001, 95% C.I. = (−0.94;-0.27); compared to 2–3 year old infants *p* ≤ 0.001, 95% C.I. = (−1.24;-0.47); compared to 3–4 year old infants *p* ≤ 0.001, 95% C.I. = (−1.13;-0.34)]. Otherwise, there were no statistically significant differences between the older age groups (comparison of 1-2-year-olds with 2-3-year-olds *p* = 0.45; comparison of 1-2-year-olds with 3-4-year-olds *p* = 0.88; comparison of 2-3-year-olds with 3-4-year-olds *p* = 0.92). Further descriptive data concerning the S-CIUS are found in Table 6.

Parents were asked about the *contexts of electronic media use* using predefined categorie (see Table 4). In the total sample of media-using children, 44.78% of children were allowed to use electronic media when parents did not have time, 37.59% of children were occupied with electronic media to calm them down, 28.36% before going to sleep, 19.12% during waiting times, 14.3% with other children and 6.06% at mealtimes. Specifically in the first year of life, media are used to occupy and calm the child (52.25%) in contrast to the following 3 years of life (42.81, 34.25, 32.02%). Additionally, the reason ‘lack of time’ shows an increase with age (16.22% in the first, 35.97% in the second, 46.79% in the third and 58.71% in the fourth year of life).

### 3.2 Multiple contextual influences

A multiple regression analysis (method enter) was used to predict *total media usage time* of all children (media and no media users) from total media usage time of all mothers, total media usage time of all fathers, parental S-CIUS-Total-score, school graduation mother, school graduation father, child gender, child age and single child versus child with siblings (Table 7). The model explained a statistically significant amount of variance in total media usage time of child, *F* (8,2,888) = 170.05, *p* < 0.001, *R^2^* = 0.32, *R^2^*_adjusted_ = 0.32. Significant predictors were: total media usage time of mother (*ß* = 0.11, *t* = 5.78, *p*≤ 0.001), S-CIUS-Total-score (*ß* = 0.05, *t* = 3.43, *p* < 0.001), school graduation mother (*ß* = −0.05, *t* = −2.45, *p* = 0.01), school graduation father (*ß* = −0.09, *t* = −4.46, *p* < 0.001), child age (*ß* = 0.56, *t* = 34.20, *p* < 0.001.) and siblings (*ß* = −0.04, *t* = −2.65, *p* < 0.01). Therefore, the final predictive model was: Total media usage time of child = 11.40 + 0.03 (total media usage time of mother) + 0.60 (S-CIUS-Total-score) −1.41 (school graduation mother) −2.28 (school graduation father) + 1.28 (child age) −2.64 (siblings). Increasing maternal total media usage time, Problematic Internet Use, lower school leaving certificate of mother, lower school leaving certificate of father, increasing age and being an only child lead to higher child media usage time. The *R^2^* for the overall model indicates a substantial goodness of fit according to Cohen (59), *f^2^* = 0.47 (large effect). Child’s gender and total media usage time of father were no significant predictors of child’s electronic media usage time.

**Table 7:** Multiple linear regression analysis results (*n* = 3,035) with “total media usage time of child” (averaged over media and no media users) as criterion.

Criterion: “total media usage time of child”					
Predictors:	*B*	*SE B*	*β*	*T*	*p*
Total media usage time of mother	0.03	0.01	0.11	5.79	0.00**
Total media usage time of father	−0.00	0.00	−0.01	−0.35	0.72
S-CIUS-total-score	0.60	0.18	0.05	3.43	0.00**
School graduation mother	−1.41	0.58	−0.05	−2.45	0.01*
School graduation father	−2.28	0.51	−0.09	−4.46	0.00**
Child gender	0.21	0.96	0.00	0.22	0.83
Child age	1.28	0.04	0.56	34.20	0.00**
Siblings	−2.64	1.00	−0.04	−2.65	<0.01*

*F*(8,2,888) = 170.05, *p* ≤ 0.001, *R*^2^ = 0.32, *R*^2^_adjusted_ = 0.32. *B* represents unstandardized regression weights, *SE B* represents standard error for *B*. Beta indicates standard regression weights; *p < 0.05; **p < 0.001.

## 4 Discussion

The present study examined the digital media use and media availability in the first 4 years of life of more than 3,000 children. Young children’s media use was examined in relation to the media use of their parents, their parents’ Problematic Internet Use, the educational attainment of their parents and family composition.

The hypotheses put forward at the beginning were partly confirmed, with mothers’ media usage, level of education of mother and father and children’s age being relevant predictors in the assumed capacities. There are no significant gender differences in the media use times of children at this early age. Siblings in this study are a factor that significantly diminishes young children’s media usage rather than increase it. In the first 4 years of their children’s lives, electronic screen media are used by parents comprehensively and depending on children’s age in different contexts (eating, falling asleep) and with different functions (to occupy/calm the child). In addition, we observed that parents of siblings had a higher S-CIUS score than parents of only children and that there was an increase in S-CIUS scores between parents of children under 1 year and parents of children aged 1 to 3 years.

### 4.1 Media characteristics by child

According to the results of our study, more than half of the 0- to 4-year-old children spend approximately 40 min using *electronic media* per day. However, of these 40 min of daily electronic media use, the use of music/audiobook with over 19 min makes up the largest part (main share). Listening, singing and dancing are highly-encouraged activities, which parents can offer to their children from an early age, either in person or through electronic media. Nevertheless, an average total screen time of 20.65 min per day remains for the first 4 years of life.

Ferjan Ramírez et al. (60) report 58 min of daily electronic media exposure in 6- to 24-month-old children and results of the miniKim-Studie (61) show comparable results to the present study, reporting that 2–3-year-olds spend 34 min watching TV. Additionally, 4% of 2–3-year-old use computer, console or online games at this early age. This is in line with results of the present study, reporting that about 3% of 2–3-year-olds and 6% of 3–4-year-olds use digital games. However, the results of our study reveal a deviation of the current practice from the recommendations of the American Academy of Pediatrics (AAP) (37, 62). Our study shows 18.30% media users in the first year of life and 61.45% media users in the second year (Table 3). If we look at the media usage times of only the media users, we find an average of 4.93 min daily for “child movies/series time” already in the first year of life and an average of 9.89 min daily in the second year of life. As well as an average daily total screen time of 7.20 min in the first year of life and 14.38 min in the second year of life. Even if we take into account that the total screen time in the first year of life includes an average of 2.25 min of (Video-)Calling/Skype daily and in the second year of life an average of 2.62 min of (Video-)Calling/Skype daily, these descriptive results deviate from the recommendations of the AAP. The AAP recommends completely avoiding the use of digital media (with the exception of video-chatting) for children younger than 18 months. If children between the ages of 18 and 24 months are to be introduced to digital media, it should always be with a caregiver and with quality educational digital media content (37).

Looking at *total screen time*, half of the 0- to 4-year-old children in the present study spend approximately 20 min in front of a screen per day. Trinh et al. (63) show an average screen time of 30 min for toddlers and 2 h for 3-year-olds. Kracht et al. (64) report 1 h of screen time per day for 3-month-olds, 1.1 h for 12-month-olds and 1.7 h for 2-year-olds. Tandon et al. (65) found much higher numbers, whereby weekday screen time for preschool children was 4 h per day, in line with the findings of Cheng et al. (66). Tandon et al. (65) points out that the usage times in the nursery/kindergarten and especially in home-based childcare should not be underestimated. As we can see in the present study, 6.07% of the children already use media in the nursery/kindergarten. Since we did not measure the time spent with media in the nursery/kindergarten, we cannot compare it with the results of the study by Tandon et al. (65). However, this shows that media time in the nursery/kindergarten cannot be neglected as it could be one of the reasons for the observed lower usage times in our sample and has the potential to become a significant additional source in the cumulative daily screen time of young children in the future.

By the end of the first year of life, approximately one fifth (18.3% of the children in our sample) are already media users. Durham et al. (67) find much higher frequencies with 45% of children already interacting with digital media in their first year of life. Kiliç et al. (68) report an average age of 12 months for the first use of mobile devices. In the present study, the frequency of media use increases sharply in the second (61.45%) and third (91.58%) years of life and reaches almost full coverage in the fourth year of life at 97.59%. There are considerable increases especially in the second year of life (by more than 40%) and in the third year of life (by about 30%). Significant course settings in media use seem to take place in the early childhood years.

With increasing age, 0- to 4-year-old children in this study are reported to use media for an increasing amount of time per day, confirming previous findings of age being a predictor of media usage time [e.g., (47, 69)]. Certain and Kahn (70) found that 83% of 0 to 11-month-olds spend less than an hour a day watching TV while 48% of 12- to 23-month-olds spend at least 1 h every day watching TV. Among the 24 to 35 months old, 16% were reported to watch 5 or more hours of TV every day, while 41% of this age group were reported to watch at least 3 or more hours daily. This finding is in line with Duch et al. (24) noting that older children (about 36 months old) have a higher screen time than younger children. As children grow older, they gain more autonomy and independence, possibly to use media by themselves as well as more fine and gross motor skills that facilitate specific and extensive media usage.

Comparisons between our data and existing studies (and between existing studies themselves) are limited by different methodological approaches (e.g., how media use is measured in the different studies or how representative the sample is).

Regarding the varying media characteristics reported in the literature, cultural differences in policy and the different policies on internet use in different countries play an important role (71, 72). In Germany, for example, internet use policies take on a crucial role, as the digitalization campaign by the German government lays the framework for a substantial increase in the use of digital media, especially in the context of schools (73).

Parents were asked in which *contexts* electronic media are used in the first 4 years of life. Results show that media are mainly used to occupy the child, especially when parents do not have time or want to calm the child down, but also before falling asleep, during waiting times, with other children and at mealtimes. This is supported by the findings of Kabali et al. (74) and consistent with findings by Vandewater et al. (9). The results of the present study contrast again with recommendations from the AAP (37), emphasizing that media should not be used to distract the child. In addition, screens are to be turned off at least 1 hour before bedtime (75). Furthermore, mealtimes and parent–child times should be media-free times. Ventura et al. (76) raise the question of whether maternal use of digital media during infant feeding has a negative impact and found that there was a negative association with some aspects of the quality of feeding interaction. In our study, during the first years of life, electronic media are used especially to occupy and calm the child. As children get older, media were used more often when parents do not have time. When it comes to media use during mealtime, we found an inverted U-shaped relationship. It seems that at the time of learning to eat independently (second and third year of life), electronic media are used particularly intensively.

In the present study, no significant *gender differences* were found with regard to screen and media use time. Consistent with previous research on older children [e.g., (53, 77)], this sample of younger children also indicates a tendency for boys to spend more time playing digital games. Girls, on the other hand, spent more time with digital picture books, which is in line with the finding of Jabbar & Warraich (78) reporting that girls are more frequent readers than boys. As girls get older, there is a higher preference for Social Media use in adolescence than in boys (77), and some studies also report more Problematic Internet Use in girls than in boys (16). To summarize, on the one hand, there is a tendency and direction toward these gender differences known from studies of older children. On the other hand, these gender-specific findings in the present study are not statistically significant. Thus, one could conclude that gender differences are not yet so pronounced at this early age. However, there is a lack of research and comparable studies on infants and young children on this topic.

### 4.2 Family context factors

The aim of the present study was to find predictors for the media use of young children, looking more closely at family factors, as there is a lack of studies for infants and toddlers in this research area. In line with prior research identifying parental media usage as a strong predictor for children’s digital media use [e.g., (41, 79, 80)], it was found that one of the major predictors in the sample of 0- to 4-year-old children was *maternal media usage*. Children spend more time watching television, playing video games and generally using screens, when their parents have a higher media consumption themselves [e.g., (42, 81)]. Woodard and Gridina (82) note that this applies especially for those parents who are heavier media users. If, for instance, a parent spends more than 2 h per weekday watching television, young children have been found to be at least 3.4 times more likely to also spend more than 2 h watching TV (83). Durham et al. (67) also point out that family TV time is a major predictor of infant screen time. In previous studies specifically mothers’ screen time (e.g., watching TV) has been found to predict the time young children spend in front of screens or engaging in media (24). The positive association between maternal media use and children’s media usage might be attributed to the fact that children learn their behavior by observing their caregivers’ interaction with the world (41, 84), as described in Bandura’s theory of social learning (85). In addition, parents’ attitudes toward the effects of media use also play a major role here, as these affect and shape the way in which parents value media in their homes (84). Parents who perceive media use as less harmful to their children may also be more inclined to expose them to more media devices more often.

In our study, the extent of *paternal media usage* was not a significant predictor of the child’s screen time. So far there has been little to no research finding comparable results for fathers when looked at outside of a parental dyad and their media usage. This relative lack of literature investigating the paternal influence on children’s media usage might be caused by mothers spending more time caring for, interacting with and even just being in the presence of their child than fathers, whose time with their children is often mediated by the presence of the mother (86). This is especially true for the first 2 years of life, when the mother plays a very significant role in parent–child interaction and - at least in the traditional model still predominant in Germany - fathers are less involved. The mother’s media consumption seems to be a significant influencing factor for the child’s media use in the first years of life, while the father’s is not. This implies that mothers are an important target group for early prevention. Kiliç et al. (68) for example showed that there is a great lack of knowledge about the effect of mobile devices: 95% of the parents who participated in their study reported that they have not been informed about the effect of mobile devices on their children by a doctor. Universal prevention programs for mothers during pregnancy and the newborn period could be implemented to share information about possible adverse effects of maternal media use. At the same time, for mothers as the main caregivers of very young children in most Western societies, there are also opportunities in the use of screens, namely to counteract the dangers of social exclusion (87) through the use of social networks. However, it is certainly favorable if this does not happen during mother–child interaction.

In addition to the mother’s screen time, the *parents*’ *Problematic Internet Use (PIU)* also plays a significant role in its effects on the child’s media use time, which is in line with the findings of Hefner et al. (88). The positive prediction power of parents’ PIU possibly indicates that parents who use the internet problematically also fail to see the dangerous consequences of digital media use for their young children, which is why their children’s media usage time might not be a (big) concern for them.

Looking at the difference in S-CIUS scores between *parents with more than one child and parents with only children*, in the present study we found that parents with more than one child have higher S-CIUS scores, indicating more PIU. To the best of our knowledge there are no studies on this topic in the current literature. Possible explanations could be that parents of multiple children have more time to use digital media because the children are engaged with each other and require less attention from parents. It could also be that the use of digital media serves as an emotion regulation strategy (89, 90) or as a coping mechanism (89, 91) due to for example increased stress caused by multiple children [e.g., (92)]. Another explanation might be that parents of several children have less opportunities for activities outside the home.

In addition, the present study found, that the *S-CIUS score of parents of 1- to 4-year-olds* increases sharply compared to the score of parents of under one-year olds. PIU of parents was significantly lower in the first year of life than in the 2 to 4 years of life of their children, increasing sharply in the second year of life. It can be speculated that there is less time and/or need to develop a PIU in the first year of life. Further studies are warranted to confirm this finding and to investigate possible mechanisms and explanations.

The present study shows that maternal and paternal *education level* significantly predicts children’s media usage time. Children of parents with higher education levels spend less time using media than children of non-academic parents, which is in line with findings of Anand and Krosnick (47) and Kiliç et al. (68). More educated parents reported less leisure media usage (81) and higher family income was negatively associated with parental media use as well (93). This is in line with the findings of Rey-López et al. (94), noting that not only parental education but also occupation influences time spent watching television. Looking specifically at maternal education, almost double the amount of mothers who had not graduated high school than of mothers who were college graduates reported that their 2 year olds watched at least 3 h of television a day (70). A woman who had not graduated from high school was almost 4 times as likely as a woman who had graduated from college to report that her 0- to 11-month-old watched at least 1 h of television per day. Overall, families with young children who have a comparatively high or even very high media intake are significantly lower educated and have a lower annual income than families who report a moderate or low usage of media. This could stem from parents with a higher level of education being more knowledgeable and educated about adverse effects of early life media usage and also being more likely to seek advice from doctors (95). Therefore, parents with a higher socioeconomic status might establish more and stricter rules regulating their children’s media and screen time and might develop these rules in a participatory joint conversation with their children (96), which could lead to a sustainable pursuit of these rules. Sebre et al. (97) highlight the importance of rules regarding social media use noting that reported rules for internet use by children were linked to lower ratings of problematic use of the internet. Additionally, parents with a higher socioeconomic status appear to be providing a reduced availability of media devices to their children compared to lower educated parents as Nikken and Schols already found in 2015 (42). Kabali et al. (74) report that young children in an urban, low-income, minority community had almost universal exposure to mobile devices, and most had their own device by the age 4. Furthermore Tandon et al. (44) note that children from lower income households are provided with a greater access to media in their bedroom and at the same time have lower access to other play equipment which promote physical activities, such as for example bikes.

In contrast Mollborn et al. (98) found that higher-socioeconomic children spend a similar amount of time with digital media devices to other groups and at the same time do not have more rules, than children from socio-economically disadvantaged families, regarding the use of digital media. This contradicts previous findings. One possible explanation the authors refer to is a theroretical perspective stating that more “advantaged” parents tend to follow an “individualistic parenting approach” (99).

When looking at specific types of media usage, Anand and Krosnick (47) found that children with fathers who either had some college education or who were college graduates were shown to spend more time using computers than children whose fathers had no high school education. This pattern is also evident in relation to playing video games or watching DVDs/videos. This could raise the question if and how various types of media usage differ and how they might be predicted or influenced by varying factors to varying degrees.

The results of our study, like those of many others state that low parental education and a low socioeconomic status are associated with children spending more time watching TV [e.g., (70, 100)]. Mollborn et al. (98) confirm this finding and note that children brought up by a college-educated primary caregiver spend less time watching TV, but more time with non-TV technology. In conclusion, one could assume that a poorer level of education could be passed on transgenerationally to the children of these families through more intensive exposure to screen media.

The present study found that *siblings* turned out to be a protective factor regarding media usage time, having siblings decreased the daily average time spend on media. As for why this is the case, it can be speculated that children who are and have siblings spend part of their leisure time with their sibling(s) instead of using media. This is in line with the findings of Bagley et al. (51) and Davies and Gentile (101). However, there are also other studies that show the opposite: Hardy et al. (102) for example found that the presence of siblings increased the time spend watching TV. The presence of other people, including siblings, during screen time is a contextual feature and thus a situational influence that could affect young children’s media-related behavior (103). As children often spend a lot of time with their siblings, even more so than with their parents [McHale and Crouter (104) as quoted in Davies and Gentile (101)] it is highly relevant to conduct further research on topics such as the potential function of the sibling as a role model, the effects of age differences between siblings, their impact on media use, and the effects of sharing digital devices (101).

## 5 Strengths and limitations

The inclusion of a relatively large data set of infants and toddlers and their parents offered the possibility to relate the children’s media use to that of their parents. In addition, other family factors such as the parents’ level of education and the number of siblings were included. The recording of media time for the child as well as for the mother and father was not done as a total value, but very differentiated according to individual categories [e.g., (Video-)Calling/Skype, internet, movies, games, picture books, audiobook]. These are all strengths of this study and extend the current literature.

As for limitations, the present study is a cross-sectional study identifying correlations, but ultimately no causal relationships. Nevertheless, we consider it more likely that family factors such as parents’ media use time, income or parental PIU score have an impact on the very young child’s media use time rather than vice versa. However, it seems that eventually there are reciprocal relationships, thus the parental PIU is lower in the first year of the child’s life than in the 2nd-4th year of the child’s life.

Critically it was only recorded whether children watch television and whether there is a television in the house, but not how much television is watched (only generally “watch movies/series”). Therefore, it is problematic to distinguish whether the time spent watching films and series is spent on the television or perhaps on the computer, smartphone or tablet, which makes it difficult to compare the present results with other studies. Similarly, the item “playing digital games” did not distinguish between educational and non-educational games. However, at present there is little research distinguishing „high-quality” (37) educational games versus non-educational games.

With 49% of both parents having a university degree and over 90% of the children living with their mother and father, the question arises as to the representativeness of the sample studied (even though it is very large). It could be assumed that more educationally distant family systems would tend to result in higher media use times.

The questionnaires used in the present study were self-report questionnaire which could lead to response biases such as under- and over-statements, as well as socially desirable answers. Furthermore, only the parents filled in the questionnaire, so there are no other data sources. Parents’ perceptions of their children’s time spent using media may be biased, inaccurate and underestimated, especially for parents with high S-CIUS scores as they may have no insight into their own or their children’s problematic behavior. Additionally, PIU was assessed only using 5 out of the 14 original items with the short version of the CIUS (S-CIUS) (56). Another issue is that the socio-economic status of the parents was only measured through educational attainment and not through further factors such as income, profession, or resources in the household.

## 6 Conclusion and outlook

In conclusion, this study yields indications for a possible problematic media consumption in early childhood in respect of the high percentage of media use in early childhood (51.99%), the average total daily screen time (20.65 min) and the context (e.g., pacifying in absence of parental resources even before bedtime) of media use. In light of the results of the present study, it is important to keep the plentiful adverse effects of media consumption in very early childhood in mind, such as negative repercussions on social, emotional, cognitive, verbal and motor skill development as well as nutrition and sleep (17–22, 81, 104–106). Excessive use of digital media can also lead to the neglect and abandonment of activities like physical exercise (107). However, some studies fail to find a negative effect (108, 109). As for the possible positive effects of using digital media at a young age, there is currently little evidence (110, 111). The AAP recommends parental interaction with the child during media use in order to provide support and guidance and to prevent excessive digital media use (37).

Because of the high educational level of the study population and the fact, that low education is correlated with high media consumption this study is very likely to underestimate the situation in the normal population. Preventive efforts to reduce the use of digital media especially among infants and toddlers seem mandatory, as early life is potentially highly relevant for further media socialization, as well as the family. There is a risk that kindergartens are playing an increasingly important part in digital socialization, however, they could also be targeted as a starting point for prevention. From the data of our study, first conclusions for prevention strategies may be drawn. The role model function of parents has to play a central role, access by the less educated population has to be assured and communication programs through pediatric practitioners should be established. Overall, this seems to be of particular relevance in order to compensate for the plethora of adversities encountered by socially disadvantaged children. Recognizing that media are a potential mediator for the transgenerational transmission of educational attainment (and ultimately Socioeconomic status SES) offers further starting points for specifically tailored indicated prevention programs.

Future research should focus on longitudinal studies to examine possible reciprocal relationships between parental PIU and the age of the child, as well as consider age of the child as a moderating factor in the relationship between parental PIU and child media use. In addition, a broader range of participants with a more diverse parental educational background as well as different living circumstances (e.g., lives with the mother and her new partner, lives with father) is needed. Regarding the contradictory findings on siblings, more research is needed on topics such as the influence of siblings’ age, the impact of sharing digital devices and also possible gender effects. In general, there is a need for more studies on infants and toddlers on the topic of digital media.

The qualitative criteria mentioned for PIU or IA or GD of adults are not transferable to toddlers and infants, for whom primarily quantitative time criteria are recorded. However, pure screen time, which was used as a quantitative measure in this study and in many other studies, does not appear to be sufficient. Future research should develop the qualitative structure and criteria of dysfunctional and disturbed media consumption in infants and children beyond the time of use.

A next step would then be to include corresponding age-appropriate criteria in the DC:0–5 (112). [The *Diagnostic Classification of Mental Health and Developmental Disorders of Infancy and Early Childhood* (DC:0–5) is a multiaxial classification system for mental disorders in early childhood providing a framework for standardizing clinical practice and research (113)].

Qualitative criteria for screen use such as educationally valuable applications, age appropriateness of the programs and level of quality of the programs need to be considered and researched more extensively. Only then will we be able to better understand what really happens during children’s screen time and how screens ultimately affect children’s development and parent–child interactions.

## Data availability statement

The datasets presented in this article are not readily available because the data that support the findings of this study are available from VM (Munich) upon reasonable request. Requests to access the datasets should be directed to VM, Volker.Mall@kbo.de.

## Ethics statement

The studies involving humans were approved by Ethics committee of the Technical University of Munich (No. 278/18S-AS; date 16.08.2018). The studies were conducted in accordance with the local legislation and institutional requirements. The participants provided their written informed consent to participate in this study.

## Author contributions

FP: Conceptualization, Data curation, Methodology, Supervision, Visualization, Writing – original draft, Writing – review & editing. JJ: Data curation, Formal analysis, Methodology, Software, Visualization, Writing – original draft. AF: Data curation, Investigation, Project administration, Resources, Validation, Writing – review & editing. TF: Data curation, Investigation, Project administration, Resources, Writing – review & editing. EM: Supervision, Writing – review & editing. VM: Conceptualization, Investigation, Methodology, Project administration, Supervision, Writing – review & editing.
